# Primary care contact before and after emergency hospitalisation in children in English NHS hospitals: a linked administrative data study

**DOI:** 10.23889/ijpds.v1i1.362

**Published:** 2017-04-19

**Authors:** Linda Wijlaars, Pia Hardelid, Ruth Gilbert

**Affiliations:** 1 University College London Institute of Child Health

## Objectives

Emergency admissions for ambulatory care sensitive conditions (ACSCs) are thought to be preventable through preventive or early treatment interventions in primary care. However, evidence shows up to 70% of children with ACSCs admissions have underlying chronic conditions, suggesting that these admissions could be less amenable to primary care prevention than previously thought. We determined the extent to which primary care is involved in care by assessing primary care consultations before and after an emergency admission to hospital for an ACSC.

## Approach

We used a national general practice database (Clinical Practice Research Datalink) linked to hospital admissions data (Hospital Episode Statistics) and included children aged 0-19 years with emergency admissions for ACSCs or injury between 2000-2009. ACSCs were defined as acute infections (pneumonia and lower respiratory tract infections (LRTIs), dehydration and gastroenteritis (DGE), urinary tract infections (UTIs)), and asthma. We included injuries as a control group as we did not expect to see an increase in GP consultations for this group before emergency admission.

We calculated hospital admission rates by age, gender and area-level deprivation per 1,000 child-years and determined the proportion of children consulting their GP in the week before and week after emergency admission. We examined independent risk factors using zero-inflated negative binomial regression models. 

## Results

We extracted data for 1,664,555 children with 32,442 emergency admissions for ACSCs and 39,305 for injuries. There were clear socioeconomic gradients: LRTIs, UTIs and asthma were more prevalent in more deprived children, while rates for DGE and injuries were similar between deprivation quintiles.

The majority of children with emergency admissions for ACSCs consulted their GP in the week before admission (range 58.3% (asthma) - 69.5% (LRTIs)), while 24.2% of children with injuries had a GP consultation. Children with ACSCs were more likely to consult their GP after discharge, with 37% (LRTIs) - 46% (UTI) of children consulting in the week after discharge, compared to 22% after injury admission.

The proportion of children consulting their GP in the week before an emergency admission decreased. For LRTIs, this decreased from 71.7% in 2000 to 55.2% in 2009.

## Conclusion

The majority of children consulted a GP both before and after an ACSC emergency admission. This suggest that the degree to which primary care can prevent ACSCs conditions in children might be less than previously thought. Further research is needed to determine which community or hospital based interventions, if any, can reduce ACSC emergency admissions.

